# Melanoma of the Hand: Current Practice and New Frontiers

**DOI:** 10.3390/healthcare2010125

**Published:** 2014-03-06

**Authors:** John Brad Turner, Brian Rinker

**Affiliations:** Division of Plastic Surgery, University of Kentucky College of Medicine, 740 S. Limestone Street, Lexington, KY 40536, USA; E-Mail: brink2@uky.edu

**Keywords:** melanoma, hand, upper extremity, sentinel lymph node, subungual

## Abstract

Melanoma of the hand represents a complicated clinical entity. Anatomic features of the hand create challenges in successful management of melanoma not encountered elsewhere in the body. The objectives of this article are to outline current standards for managing melanoma of the hand including diagnosis, surgical, and chemotherapeutic management. Particular emphasis will be placed on currently debated topics of the role of sentinel lymph node biopsy, the role of Mohs micrographic surgery, tissue sparing management of subungual melanoma, and the consideration of melanoma of the hand as a distinct entity based on clinical and molecular studies.

## 1. Introduction

Melanoma is a substantial public health problem in many countries and a devastating diagnosis for patients to receive. Melanoma is a melanocyte-derived malignancy that accounts for only 4% of cutaneous malignancy but 80% of skin cancer related deaths. According to the American Cancer Society for the year 2013 there will be an estimated 76,690 new cases resulting in 9480 deaths in the Unites States alone [1].

A diagnosis of melanoma of the hand presents several unique challenges to the healthcare team involved in management of these patients. The primary care physician or dermatologist is often the first to encounter a concerning lesion. The decision regarding the role and type of biopsy are often discussed with the patient. After diagnosis is made, referral to an appropriate surgeon, depending on lesion size, depth, and concern for long-term function of the hand is the appropriate next step in management. 

There are a variety of specialists well equipped to manage melanoma of the hand, all with varying attributes. Plastic Surgeons, Hand Surgeons and Dermatologists may be comfortable performing both local excision and amputation, along with a functional reconstruction if indicated. In many centers, however a Surgical Oncologist or General Surgeon will prefer to perform the excision as they are in general more facile with current techniques and concepts of sentinel lymph node biopsy (SLN) and nodal dissections when they are indicated. There are many cases where both groups will be needed to offer the best care to the patient from both an ablative and restorative standpoint. A general knowledge of local referral patterns and preferences of consultants will aid in expedient management of this patient population.

The purpose of this article is to provide a concise review of the recent literature from which all healthcare practitioners can gain insight into the current diagnostic and therapeutic management related to melanoma of the hand. We will highlight significant differences in melanoma of the hand relative to cutaneous melanoma in the rest of the body and underscore the need for further study given the limited evidence in the extant literature. 

## 2. General Principles of Hand Melanoma (Non-Nail Unit Lesions)

### 2.1. What Is Different about Hand Melanoma?

The cutaneous components of the hand represents only 1%–2% of the total body surface area yet harbors 10%–15% of all cutaneous malignancies [2]. This small surface area portends challenges to the surgeon who attempts to balance sound oncologic principles with the preservation of hand function. At the present time there are no published consensus guidelines for management of melanoma of the hand. Additionally, to our knowledge no tumor registries identify hand melanoma as a separate entity. This has limited our understanding of the epidemiology and outcomes of this clinical entity to mostly retrospective cohort studies and isolated population based studies. 

The incidence of cutaneous melanoma is highest in Caucasian populations. However regarding melanoma of the hand, including subungual melanoma, non-white populations are as frequently if not more often affected. Acral lentiginous melanoma is the most common type found in darkly pigmented patients [3]. 

There is a limited subcutaneous layer in the hand with underlying functional structures *i.e.*, bone and tendon, within several millimeters of the base of the dermis. There is a classic “danger zone” in the hand with regard to metastatic potential. The webspaces and skin overlying the proximal phalanges are an area of convergence of the superficial digital lymphatic plexus from both the volar and dorsal side of the hand and finger. With relatively thinner skin, this results in what is theorized as an explanation for the increased rate of metastasis of all types of cutaneous malignancy seen in these anatomic subunits [4]. 

Until recently there has been a paucity of data looking specifically at skin cancer of the hand. Maciburko *et al.* recently reported a series of 541 hand malignancies of which only 3.9% were found to be melanoma [5]. The median age was 66.5 years with a slight female predominance. The authors found 61.5% to be acral lentiginous, 15.4% to be superficial spreading, and 15.4% to be nodular types. When delineating the anatomic distribution of the malignancy, melanoma was most commonly seen in the palm (14%), tip/nail complex of the digit (29%), and the dorsum of the hand (47%). They noted that most of their patients had T3 (2.01 to 4.00 mm thickness) lesions upon presentation and that the excision margin was less than recommended based on guidelines published late in their study period. Metastasis to regional nodes was found in 50% of the patients. Despite the proportion of patients with advanced disease at diagnosis, the 5-year survival was 76.2% for the study period. There is a theme throughout the literature that hand melanoma may be a more “aggressive” form. One can certainly deduce from this study that late diagnosis is a significant contribution. The true “aggressiveness” of hand melanoma, many of which are the acral lentiginous type, will only be elucidated with future multicenter studies [5,6]. At the current time anatomic location is not an established prognostic indicator, care should be taken in interpretation of the results of this study. However, this may be indicative of a new frontier in the field of melanoma.

In a systematic review of hand and foot melanoma (HFM) Durbec *et al*. note several differences compared to general cutaneous melanoma [7]. UV radiation is likely not to play a significant role as a risk factor for the melanoma of the palm and sole but previous trauma and acral nevi may be a significant risk factor not previously entertained. Sentinel lymph node biopsy has not been adequately evaluated as a means of prognostication in HFM. The authors also note a significant difference in genetic features between HFM and other melanomas. In general, HFM has a higher percentage of c-kit + tumors and fewer with BRAF mutations. These differences will have significance as the role of molecular therapy in melanoma increases with further study and advent of novel drugs, such as tyrosine kinase inhibitors [7]. This new information represents a new avenue in the management of hand melanoma. 

### 2.2. Diagnosis

For lesions not involving the fingernail complex, the ABCDE system of describing cutaneous melanoma has applicability in the hand. Asymmetry of the lesion, irregular Borders, Color variation within the lesion, Diameter >6 mm, and Evolution over time are all concerning features in pigmented lesions. Once a concerning lesion is identified the only way to rule out malignancy is with a tissue diagnosis. 

In an effort to minimize the number of unnecessary biopsies of suspicious lesions adjunct diagnostic methods have been studied. Epiluminescence microscopy or dermoscopy is a method of evaluating a lesion *in-situ*, with the aid of a magnifying lens. There are many models available with varying properties. In well trained hands, the addition of dermoscopy in clinical evaluation of pigmented lesion has increased the sensitivity relative to the unaided eye by up to 27% [8]. The dermoscope is beneficial in delineating nail unit melanoma as well. A newer skin imaging modality includes *in-vivo* confocal laser scanning microscopy. This modality utilizes laser reflectance to evaluate skin structures and has demonstrated an 86.1% sensitivity and 95.3% specificity in diagnosing melanoma [9].

Breslow depth of melanoma (from the granular layer of the epidermis to the deepest atypical melanocyte) is the most cited measurement and foundation upon which all treatment plans are made [10]. This should convey the importance of a quality full thickness biopsy. Incisional, excisional and punch biopsy techniques may all be used depending on characteristics of the lesion and physician comfort with each technique [3]. Partial thickness biopsy, also known as a shave biopsy, should not be used if the diagnosis of melanoma is within the differential. 

The current standard techniques for histologic evaluation are Hematoxylin and Eosin stains with additional stains of MART-1/Melan-A, S-100 and HMB-45, as needed. There are many architectural features shared between early melanoma and other benign pigmented lesions, are documented in the pathology literature. The current recommendation is to seek the opinion of a second certified pathologist for any questionable lesions [11].

If deeper invasion of the tumor is concerning on clinical exam, magnetic resonance imaging should be considered. While no clinical guidelines exist due to the innumerable variables involved, the ability of MRI to delineate invasion and proximity to underlying bone, tendon, nerve, or vessels may aid in preoperative planning with regard to ablative planning and reconstructive options. Imaging may alert the specialist if an amputation may be more functional than a prolonged reconstructive effort [3].

### 2.3. Staging and Management

Given the absence of data regarding the specific staging of melanoma of the hand staging should be performed in concordance with the most recent institutional guidelines. The lesion should be assessed for greatest Breslow thickness, Clark level, mitotic index, presence of lymphocytes, microsatellites, and ulceration. Regional nodes should be evaluated by physical examination and clinically concerning nodal basins should be sampled with a surgical procedure [12]. Based on clinical symptoms, a metastatic workup can be initiated with chest x-ray and liver function tests being the most fundamental. The most commonly accepted staging system at our institution is the TNM classification outlined by the American Joint Commission on Cancer 7th edition. 

#### 2.3.1. Margins of Excision

Surgical margins for the safest excision of melanoma have come under scrutiny in recent decades. From the time of Handley in the 1900’s up until the 1970’s recommended margins of resections were 5 cm based on necropsy specimens and early reports of “field changes” of resulting atypia of melanocytes up to 5 cm from a lesion [13]. Since that time there have been over 200 studies examining safety of excision margin in melanoma. Recommendations for surgical margins have been relatively unchanged in the last decade when multiple consensus clinical guidelines were released (see Table 1). A discussion of recent literature touting the safety of smaller margins for melanoma has not examined specifically melanoma of the hand and is beyond the scope of this article. Timing to excision can be safely delayed up to 3 months from the time of initial biopsy with no effect on survival or local recurrence should the clinical situation arise [14].

**Table 1:** Recommended excision margin.

Depth of Lesion	World Health Organization Trial [15]	Australian Trial [16]	Dutch Trial [17]	United Kingdom Trial [18]
*In situ*	5 mm	5 mm	5 mm	5 mm
<1 mm lesion	1 cm	1 cm	1 cm	1 cm
1 to 2 mm	1–2 cm	1 cm	1 cm	1–2 cm
2.1 to 4 mm	2 cm	1 cm	2 cm	2–3 cm
>4 mm	2 cm	2 cm	2 cm	2–3 cm

#### 2.3.2. Sentinel Lymph Node Biopsy

Sentinel lymph node biopsy has revolutionized the management of regional disease in many types of cancer. Current practice is to offer sentinel lymph node biopsy to patients with certain characteristics for patients with extremity melanoma. It is estimated that 20% of all patients with intermediate thickness melanoma (1.2 to 3.5 mm in the trial) will have a regional lymph node metastasis [19]. For those patients with microscopic nodal metastasis there is a 40% decrease in 5-year survival. Sentinel node biopsy offers the benefit of identifying those patients that stand to benefit the most from complete lymphadenectomy. SLN biopsy is performed by the injection of a radioactive colloid and dye (isosulfan, methylene blue or indocyanine green) in the dermis of the lesion. The sentinel node in either epitrochlear (in-transit) or axillary beds is then identified. Once identified with a gamma radiation detection probe, the node with the highest gamma count, any clinically dyed nodes or nodes with a dyed vessel, and those at 10% of the “hottest” node are removed for histologic evaluation [20,21].

The likelihood of obtaining a positive sentinel lymph node biopsy increases with increasing Breslow thickness of the lesion. For lesions 1.0 mm or less, 1.01 to 2.00 mm, 2.01 to 4.00 mm and greater than 4 mm the risks are approximately 4%, 12%, 28%, and 44%, respectively [20].

The role of SLN biopsy in melanoma has recently been investigated with the Multicenter Selective Lymphadenectomy Trial I and II (MSLT-I and MSLT-II). The MSLT-I trial has reached completion and the MSLT-II trial is still ongoing. MSLT-I did not demonstrate an overall-survival benefit for those patients undergoing SLN biopsy followed by completion lymphadenectomy compared to those receiving delayed completion lymphadenectomy. However for those patients undergoing SLN biopsy with immediate completion lymphadenectomy there was an improvement in disease-free survival and disease specific survival at 5 years [12,21]. Sentinel node biopsy has a clinical staging accuracy of at least 96% and has been shown in multiple studies, to have minimal morbidity. The complication rates vary but are usually less than 5%. Lymphedema is exceedingly rare and complications are generally limited to mild infections, small hematomas or seromas that can be resolved with conservative measures. The prognostic benefit of sentinel node biopsy, coupled with the low risk, has allowed it to remain the gold standard for regional nodal evaluation in the intermediate thickness melanoma patient. The most recent American Joint Commission on Cancer (AJCC) staging system includes SLN biopsy data [21]. There are still opponents of the technique, but until a non-invasive method of detecting microscopic nodal disease is discovered, SLN biopsy will remain an important tool in the prognosis and treatment stratification of melanoma patients.

Current recommendations for SLN biopsy are patients without clinical evidence of nodal disease with lesions >1 mm. Consideration should be made to offering patients SLN biopsy for lesions <1 mm with atypical features such as elevated mitotic rate, ulceration, or satellitosis [3]. It should be noted that this has not been studied specifically for hand melanoma. Further study is warranted given the noted clinical and genetic difference already discussed.

### 2.4. Role of Mohs Micrographic Surgery

In 2012 the American Academy of Dermatology, American College of Mohs Surgery, American Society for Dermatologic Surgery and the American Society of Mohs Surgery released a consensus guideline regarding the appropriate use of Mohs surgery. At that time the only acknowledged uses were for lentigo maligna and melanoma *in situ*. Areas of the body were stratified into groups of Area H, M, and L, of which the hand and nail unit were considered Area H. Mohs micrographic surgery was deemed an appropriate technique based on their analysis for lentigo maligna and melanoma *in situ* of the hand and nail unit [22]

Recent reports of using Mohs surgery for nail unit lesions in particular are sparse. This represents a potential new application of a known entity although certainly further study is warranted. Brodland described the successful use of the technique in 2001 [23]. There are very few case reports of its use in invasive melanoma [24]. A multitude of studies have demonstrated safety with narrower margins of melanoma. As the technique of Mohs surgery continues to evolve and gain more acceptable indications its role in management of melanoma of all types is likely to expand. 

## 3. Subungual Melanoma

### 3.1. Diagnosis

**Figure 1:**
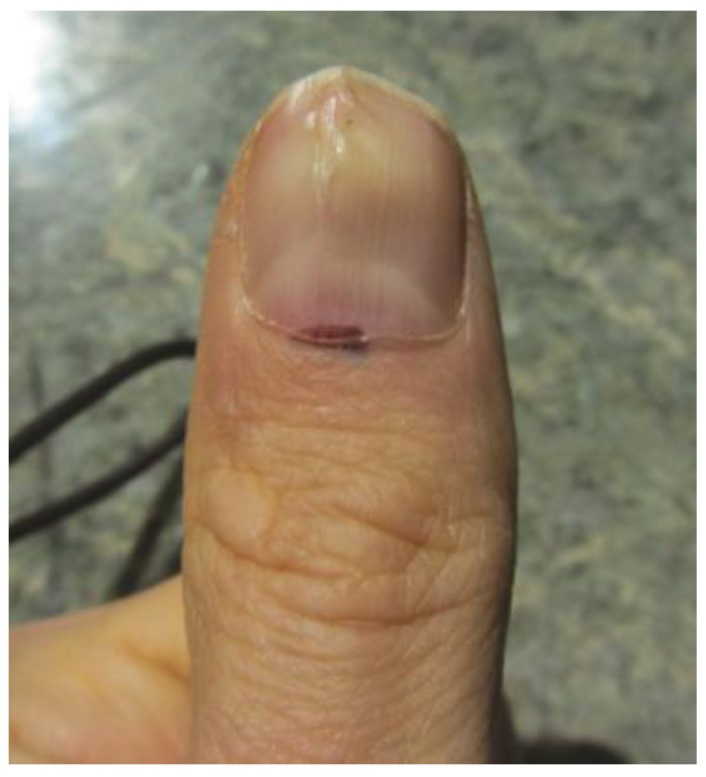
Subungual melanoma of the left thumb. Note pigmentation of the nail fold and faint pigmentation and abnormality of the nail plate.

Subungual melanoma is melanoma of the nail matrix. Boyer identified it clinically as early as 1834. Subungual melanoma is thought to affect dark-skinned populations in greater proportion and occurs most commonly in the 5th to 7th decades of life [25]. There is a predilection of the thumb [7]. It commonly presents as changes in the color of the nail plate and underlying nail bed. Ulceration, bleeding and pain occur less commonly. Pigmentation of the eponychium is a sign concerning for malignancy, this is commonly referred to as Hutchinson’s sign. Sir Jonathan Hutchinson in 1886 called it “melantotic whitlow” given its similarities with nail infections (see Figure 1) [25]. The differential, of a pigmented nail lesion does include infection as well as hematoma, chemical exposure, medication reactions, HIV, vitamin deficiencies, as well as many others [3,26].

The most common histologic type of subungual melanoma are acral lentiginous and nodular. One study demonstrated an average Breslow scale of 3.1 mm. Characteristics of the nail bed that make histologic diagnosis challenging include a poorly delineated dermis, minimal subcutaneous fat, propensity for epidermal hyperplasia and lack of an epidermal granular layer. This has led some to abandon the Breslow classification for treatment decisions, instead considering lesions limited to the matrix itself as *in situ* lesions and those full thickness to be treated as invasive often electing to perform amputation [26].

When not clinically evident, imaging of lesions of the nail unit can provide valuable information regarding the type of lesion or infiltration of deeper structures and can aid in surgical planning. Ultrasound and MRI are soft tissue imaging modalities most relevant to nail complex lesions and should be considered in equivocal cases to further aid diagnostic and surgical planning [27].

### 3.2. Staging

**Figure 2:**
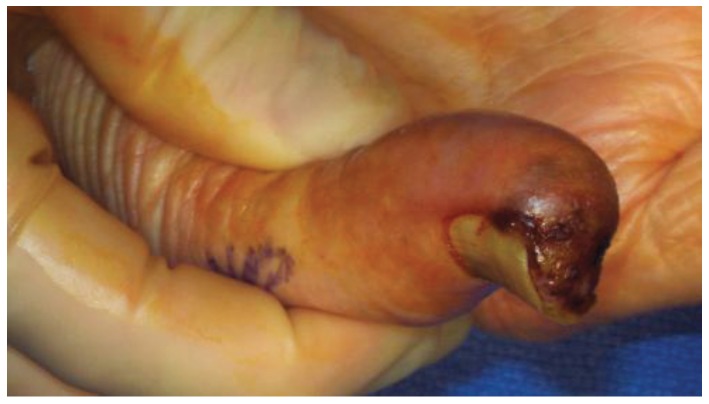
Subungual melanoma of the right thumb. Note pigmentary changes extending onto the pulp and exophytic nature of the lesion.

A high index of suspicion is needed in evaluating lesions of the nail. This has led to an adaptation of the ABCDE’s of cutaneous melanoma to its subungual counterpart. Age of the patient being in the 5th to 7th decade of life, Band of nail pigmentation greater than 3 mm or having irregular borders, Change in color or size, Digital characteristics such as being isolated pigmentation of the thumb rather than multi-digit discoloration, Extension to the periungual skin, and Family history make up the ABCDEF’s of subungual melanoma (see Figure 2) [28].

If a lesion is clinically concerning for hematoma or an onychomycotic infection, a period of no more than 8 weeks of observation and treatment should be undertaken. Once a concerning lesion is noted a full-thickness biopsy should be performed. This can be done in a number of ways. First, the patient should be anesthetized with a digital block. Often only a small portion of the nail plate can be removed, exposing the area to be biopsied without removing the entire nail unit. For physicians not familiar with nail bed repair techniques, a punch biopsy, with or without nail plate removal, can be performed. For physicians familiar with nail repair techniques a diamond shaped incisional biopsy or linear excision can be performed [26].

### 3.3. Staging and Management

**Figure 3:**
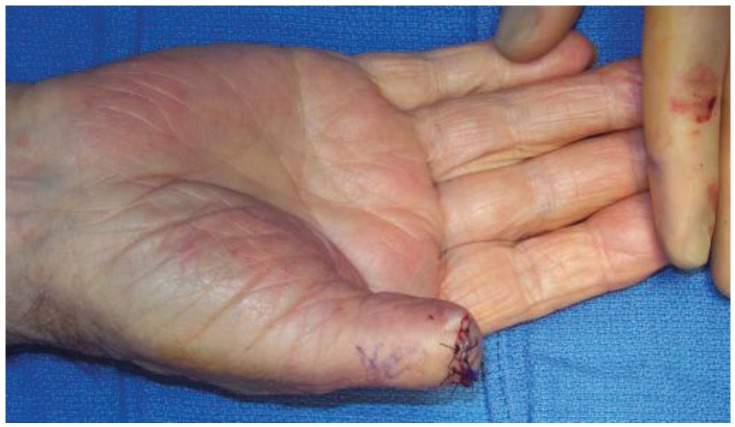
After interphalangeal joint level amputation (patient from Figure 2).

Until recent years the scarcity of data had led most surgeons to amputate any melanoma lesions of the nail complex. Puhaindran and colleagues evaluated their results for management of malignant tumor of the thumb. They utilized the Musculoskeletal Tumor Society Score (MTSS), which examines the emotional, functional, and pain aspects of tumor management. They found that patients undergoing interphalangeal joint amputation faired equally as well as those undergoing thumb-sparing surgeries. Amputation is a well-tolerated procedure if length can be preserved (see Figure 3). They did note that amputations at the level of the metacarpophalangeal joint are poorly tolerated [29].

In 2003 Moehrle and colleagues described their outcomes with “functional” surgery for subungual melanoma. It consisted of local excision of the lesion with partial resection of the distal phalanx and reconstruction with full thickness skin graft. With 31 patients in each group they found no differences in survival or recurrence free survival [30]. This represents a method that while not “new” has yet to gain widespread consideration as a method of treatment. 

Yun and colleagues recently described their experience with 27 hand melanomas over an 18-year period. Their population was slightly younger, and most lesions were localized to the digits having no dorsal hand lesions in the series. Seventeen of the 27 lesions were T1 lesions or less. Amputation was the most commonly performed procedure, 13 of 27 cases, but as their experience grew they employed less aggressive resections. Ultimately, only 1 of the 27 cases resulted in a recurrence/metastatis. The authors surmise that the anatomy of the fingertip, with its multilayered complex and absence of conduits such as hair follicles, contributed to the paucity of advanced disease. Whether this is a selection bias given the number of low-grade lesions or whether their theory is correct will require further investigation [31].

The limitations of these studies are apparent. Many are retrospective and small cohorts so care should be taken in applying their interpretation into clinical practice. Patients should be counseled thoroughly before embarking on any conservative melanoma resections.

### 3.4. Prognostic Data

Prognosis is generally thought to be poor. Nguyen *et al*. note in their series a 5-year recurrence free survival of 57.1% and 5 year survival of 60.5% for all patients. As with all type of cancer, early detection and biopsy of concerning lesions are the keys to a favorable outcome. With regard to subungual melanoma we have already discussed the challenges with histologic diagnosis. Until further evidence is brought to bear on the subject, SLN biopsy should be offered to all patients with invasive subungual melanoma for staging of regional nodes [26].

## 4. Surgical Management

### 4.1. Reconstructive Goals in Hand Surgery

The hand is an extension of the human brain, without it the civilization we know today would be precluded from existence. The hand is unique in many ways. There are 5 digits each with relatively different functions. The thumb is given greatest value, when absent the hand is considered to have lost 40% of its inherent function. The remaining digits including index and long fingers are primarily used for fine motor tasks, with great mobility and minute manipulation. The ring and small finger are in a location that is mechanically advantageous and are designed to perform tasks requiring composite grip and strength.

The volar and dorsal sides of the hand are also unique. Differences in this soft tissue envelope, which is our primary concern after ablation of melanoma, are readily apparent and convey functional disparities between the sides. The volar side, which is the working surface of the hand, requires tough, adaptable, and highly sensate skin. Dorsally, the skin is mobile, elastic and expandable allowing full range of motion over the convex surface. 

The goals of soft tissue reconstruction in the hand should be to replace what has been removed with tissue that is as identical as possible. One can easily infer that the hand is an area of the body with a relative paucity of skin. A lesion of 6 mm diameter and Breslow thickness of 1 mm requires a minimum of 2.6 mm diameter skin excision. On the dorsum of the finger this encompasses a large portion of the circumference and can be function limiting if not appropriately reconstructed. There are instances where the recommended margin may not be able to be performed. It is thus prudent to spend time counseling the patient beforehand.

### 4.2. Soft Tissue Reconstruction

Replacing “like with like” is a mantra in field of Reconstructive Surgery. Goals of reconstructing soft tissue of the hand include providing durable, sensate, aesthetically sound tissue with consideration of donor site morbidity, patient demands and comorbidities. Additional goals of preserving bony length, maintaining joint function and minimizing the number of reconstructive stages are also considered. 

Primary closure, re-approximation of wound edges with suture, is the most desirable form of reconstruction. This is often possible on the trunk or proximal extremities but not feasible in the hand or fingers. If a wound is unable to be closed then the surgeon can consider healing by secondary intent. This is often employed in the fingertip where moist dressing changes will allow contraction of the wound periphery bringing in sensate, glabrous skin for potentially stable reconstruction. This method requires a viable wound bed without bone or tendon exposure in order to be a viable option. The disadvantage is that the wounds can take weeks to heal and may be uncomfortable in the interim. 

A skin graft is can be utilized in reconstructing defects that cannot be closed with either of the prior methods. There are 2 broad categories of skin graft. Full thickness skin grafts are generally an ellipse of skin removed from the donor site, which can be closed primarily leaving a linear scar. The graft has the entire thickness of dermis, which resists contraction of the graft, and recipient wound bed. Full thickness grafts are utilized throughout both the volar and dorsal surfaces of the hand due to this property. Common donor sites include the hypothenar eminence, volar wrist crease, groin, and medial arm. Each has various size and limitations with regard to color match at the recipient site. A split thickness skin graft can be utilized in the hand though generally reserved for very large surface areas for which a full thickness graft of appropriate size would not be possible. Split thickness skin grafts are taken from the donor site with a dermatome, which ultimately must heal by secondary intent from the dermal appendages and wound edges. The grafts are then secured to the recipient site in a number of ways but are generally unmeshed in order to minimize contracture. Despite these efforts split-grafts are prone to contracture and the donor site is uncomfortable during the healing process. 

When a defect on the hand has involved or exposed any of the vital structures including, bone, joint, tendon, nerves or vessels, grafting techniques are not possible. A flap is composite vascularized tissue that can be transferred into a defect bringing its own blood supply and must be considered in this situation. It does not rely on the underlying bed for healing. Flaps can be composed of many tissues including skin, fat, fascia, muscle, bone, tendon, nerve, vessels or any combination of the above. The flap is designed to deliver “like tissue” to the area. The primary disadvantage of a flap is that a deficit is created at the donor site and that morbidity should be weight against the benefit.

**Figure 4:**
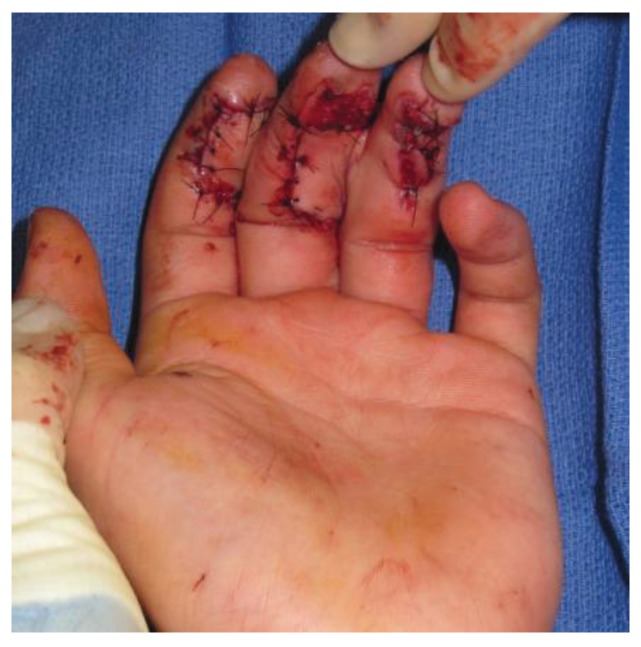
Cross finger flaps used for closure of traumatic defects of the index and long fingers.

**Figure 5:**
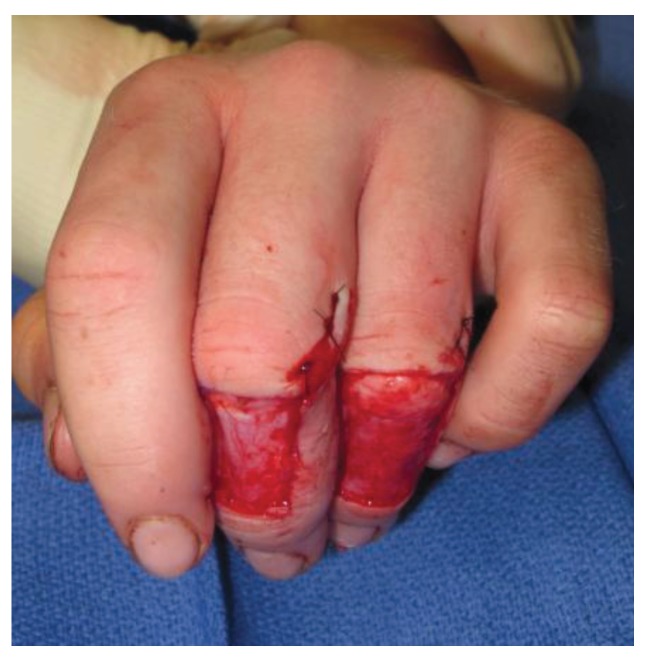
Donor site of the cross finger flaps prior to application of a full thickness skin graft.

The litany of flaps used to resurface defects of the hand is vast and both the number and modifications to existing flaps continue to increase. There are several worth mentioning. In the fingers and thumb there are often few options for local flaps. Often skin must be mobilized on the same digit, homodigital flaps, with the best example being the Moberg flap for thumb tip reconstruction. The soft tissue of the thumb is mobilized on both radial and ulnar neurovascular bundles and advanced to the tip for small defects. When local options are not available a nearby digit may be utilized. The cross finger flap borrows either skin or the adipofascial layer of the dorsum of the hand for coverage of a neighboring digits defect (see Figure 4 and Figure 5). A skin graft often must be placed as well for the donor site and the digits are temporarily bridged together while the recipient vascularizes the flap periphery [32].

**Figure 6:**
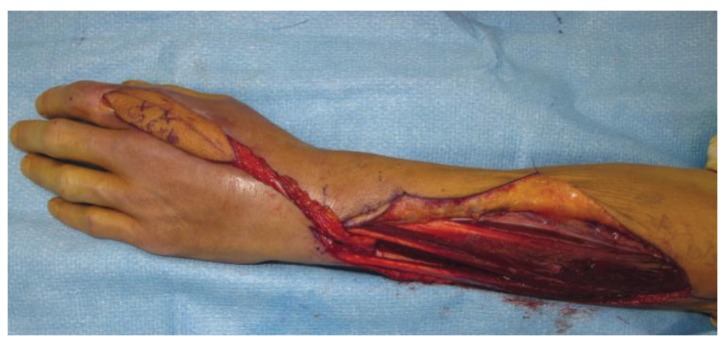
A dissected reverse posterior interosseus artery flap for reconstruction of a dorsal hand defect with exposed tendon after infection. Not the pedicle based at the distal forearm.

For large defects of the dorsum of the hand, flaps from the forearm remain a mainstay in management. The reverse radial forearm flap is a flap based on retrograde flow through the palmar arch allowing tissue from the forearm to be transposed to a variety of locations. The donor site can be closed primarily or with a graft. Alternate forearm based flaps include the reverse posterior interosseous artery flap which has similar indications but does not sacrifice the radial artery of the patient (see Figure 6) [33].

A final reconstructive modality to mention is free tissue transfer. This form of reconstructive surgery requires expertise in many areas particularly that of microvascular surgery. In a free tissue transfer procedure a flap is designed about an artery and vein to a particular angiosome of tissue. It is then transferred to the recipient site where microvascular anastomosis is completed to establish blood flow to the flap. The advantage of free tissue transfer is that the surgeon can be more selective in the type of tissue transferred whether it be for the addition of bone to the flap or necessitating a more reliable and thinner flap. Common free flaps employed in reconstructing the hand include the lateral arm flap, anterolateral thigh flap, temporoparietal fascia flap with skin grafts, and many others [32]. 

The type of reconstructive procedure utilized for a tumor defect is dependent upon numerous factors. For any particular defect there will be multiple methods of reconstruction. The challenge for the reconstructive surgeon is finding what best suits the needs of the patient. 

## 5. Adjunct Therapy

### 5.1. General Considerations

Consensus on the most clinically effective methods of managing late stage (III and IV) melanoma of the hand is not available at this time owing in large part to the relative infrequency of occurrence. Cohort studies of advanced cases of melanoma of the hand are ongoing. Currently accepted treatments include High Dose and Pegylated Inteferons for resected disease and newer medications including Ipilimumab and Vemurafenib for Stage IV and unresectable disease. Trials continue for both [34]. A comprehensive review of adjunct treatments of late stage melanoma is beyond the scope of this review but there are several treatment modalities unique to the hand and extremities worth discussion.

### 5.2. Hyperthermic Isolated Limb Perfusion and Isolated Limb Infusion

Two techniques of regional chemotherapy have been introduced which have been essentially deemed palliative. These techniques are available only at advanced centers with the capabilities and familiarity with the techniques. Hyperthermic isolated limb perfusion is a complex surgical procedure, which places the extremity on complete vascular bypass, isolated from the remainder of the body. High dose melphalan is infused and warmed to 41 °C. Complications including skin necrosis, neuropathy and compartment syndrome are frequent. Isolated limb perfusion is a less invasive technique requiring percutaneous cannulation of the artery and vein. A higher dose of chemotoxic agents are not able to be achieved nor is there a hyperthermic component due to lack of complete isolation from the systemic circulation. As would be expected, studies have demonstrated improved tolerance but less effectiveness with this technique [35].

## 6. Conclusions

As evidence suggests, melanoma of the hand is an anatomic, genetically and clinically distinct entity. Management requires cooperation from multiple medical practitioners. Future research efforts will delineate an appropriate set of guidelines for treatment of melanoma of the hand.
